# Fabrication of cerebral aneurysm simulator with a desktop 3D printer

**DOI:** 10.1038/srep44301

**Published:** 2017-05-17

**Authors:** Yu Liu, Qing Gao, Song Du, ZiChen Chen, JianZhong Fu, Bing Chen, ZhenJie Liu, Yong He

**Affiliations:** 1 State Key Laboratory of Fluid Power and Mechatronic Systems, School of Mechanical Engineering, Zhejiang University, Hangzhou 310027, China; 2 Key Laboratory of 3D Printing Process and Equipment of Zhejiang Province, School of Mechanical Engineering, Zhejiang University, Hangzhou 310027, China; 3 Department of Neurosurgery, Second Affiliated Hospital, School of Medicine, Zhejiang University, Hangzhou 310009, China; 4 Department of Vascular Surgery, Second Affiliated Hospital, School of Medicine, Zhejiang University, Hangzhou, 310009, China

## Abstract

Now, more and more patients are suffering cerebral aneurysm. However, long training time limits the rapid growth of cerebrovascular neurosurgeons. Here we developed a novel cerebral aneurysm simulator which can be better represented the dynamic bulging process of cerebral aneurysm The proposed simulator features the integration of a hollow elastic vascular model, a skull model and a brain model, which can be affordably fabricated at the clinic (Fab@Clinic), under $25.00 each with the help of a low-cost desktop 3D printer. Moreover, the clinical blood flow and pulsation pressure similar to the human can be well simulated, which can be used to train the neurosurgical residents how to clip aneurysms more effectively.

Every year, the number of those who suffer from a ruptured cerebral aneurysm is approximately 10 in every 100,000 persons, while those who may have an un-ruptured cerebral aneurysm take up as high as 5 percent of the American population^1^. Permanent nerve damage, hemorrhagic stroke or even death are all serious complications that could be seen in the cases of aneurysmal rupture whose mortality could reach 45%^2^. The treatment for cerebral aneurysms is usually secured with coils or clips to prevent re-rupture, which is a ubiquitously-accepted approach. After the publication of ISAT study^3^, an increasing number of patients has received the endovascular treatment of aneurysms. According to some studies, cerebrovascular neurosurgeons still prefer surgical clipping to treat specific aneurysms^4^,^5^,^6^ depending on lesion morphology, their size and location. Therefore, the demand for developing various efficient and safe surgical training methods has risen under such a circumstance^7^. Patients and their attending surgeons are faced with a tough challenge in managing ruptured or un-ruptured cerebral aneurysms. It is always a neurosurgeon, an expert of cerebrovascular disease, that handles the surgical clipping of a cerebral aneurysm, but the neurosurgical residents are seldom offered with chances to perform and learn from surgeries in spite of the long-lasting demand for aneurysm clipping as well as the high risks related to opening neurovascular surgery. For operating cerebral aneurysm surgery, a prerequisite for a cerebrovascular neurosurgeon is a comprehensive understanding of the shape of aneurysm as well as its positional relationship with the cranial nerves, aorta and branches, brain, skull, etcetera^2^. To obtain such an understanding, cerebral angiography used to the only accessible approach for most cerebrovascular neurosurgeons. The cerebral angiography constructs a mental three dimensional images through combining a number of two dimensional images in the brain. However, the complex vascular network that neighboring the aneurysm does pose a thorny challenge to the 3D structure visualization in this method, which certainly requires a lot of training as well as sufficient experience. Usually, a five-to-seven-year general neurosurgery training plus a one-to-two-year specialized cerebrovascular training is a basic requirement for most cerebrovascular neurosurgeons. To address the challenge, learners could utilize a medical simulator with proper tactile feedback that offers a risk-free environment for them to be engaged in surgical practices and polish skills. The surgical device could simulate the real operation environment and practice facilitating the surgical education as well as the hands-on trainings^8^,^9^. Cadaveric trainings are often taken by medical students to mimic the procedural surgical training, which is also counted as the clinical experience during residency^10^,^11^.

But the cost of facilities and cadavers for surgical training keeps climbing up^12^. Under the pressure of rising costs and limited resources of cadavers, medical simulator turns out to be a cost-effective solution adopted for procedural training to gain learning and practice concerning cerebral anatomy and diseases. The finite element method has been used for the computer simulation of brain retraction and displacement^13^.

The advantages of simulation have been well established based on a full exploration into its application as a surgical training modality^14^,^15^,^16^,^17^,^18^. The benefits of simulation are widely acknowledged, especially the value in the neurosurgical residency training, according to neurosurgery residency program directors^19^. Simulators for aneurysm surgeries have already been developed previously^20^, with solid models being applied for multiple purposes like informed consent process, pre-surgical assessment and training of junior surgeons.

Recent years have witnessed an increase in the amount of related studies, such as evaluation for the clipping position simulation of cerebral aneurysms based on 3D computed tomography (CT) angiography^21^,^22^, application of 3D printing technology to generate cerebrovascular simulacra as cerebral arteries^23^, and the adoption of functional anatomy of integrated sensory signals across the visual and tactile modalities mapping by positron emission tomography^24^. 3D printing technology has been soon accepted as a patient-specific fabrication method and a cost-effective way of producing 3D objects as well as a source of novel clinical and biomedical applications^25^,^26^,^27^,^28^,^29^,^30^,^31^,^32^. 3D models also appear to be an ideal educational tool^33^ that allows the simulation and practice of diverse basic and advanced surgical procedures such as ventriculostomies^34^,^35^. However, early models used to be limited to rigid, non-hollow materials mainly. Then a manufacturing process was adopted to successfully build true-to-scale silicone arterial replicas based on silicone casts so that the wall thickness is uniform^36^. Acrylonitrile-butadiene-styrene was adopted as a modeling material along with silicone as the outer layer to produce a hollow elastic vessel model, which could receive a clip in the performances of simulations^37^. Besides, medical learners were also offered with a modular, reproducible medical simulacrum for the exercising aneurysmal clipping procedures^38^. Some simulators are still viable though the methods offered could only deal with some issues to some extent and it is believed that the author’s design and the proposed static simulators not allowing flow within vascular model could be immediately improved with the progresses in 3D printing technology.

Accordingly, we developed a cerebral aneurysm simulator using the low cost three-dimensional printer, silicone casting and coating techniques. The goal of this simulator is to deepen understand the dynamic bulging process of cerebral aneurysm and train neurosurgical residents how to clip aneurysms effectively. Towards the proposed goal, we successfully produced a skull, a silicone brain and a vascular model. The cost of each under $25.00 is certainly affordable to a lab, with no other costly technical facilities. A hollow elastic vascular silicone model as constituent part is a feature of the proposed simulator, allowing for blood flow. A skull model and a brain model that behave the neighboring positional relation and recreate the surgical reality scene are integrated. Moreover, it is proven that the proposed vascular model can simulate the dynamic bulging process of cerebral aneurysm. The blister-like bulging regularity recreates the clinical blood flow and pulsation pressure similar to the human.

## Results

### Fabrication of cerebral aneurysm simulator

In our approach, the cerebral aneurysm simulator consisting of a skull model, a brain model and a vascular model with blister-like dilation bulges was fabricated by a desktop 3D printer combined with silicone casting and coating techniques.

**Figure 1:**
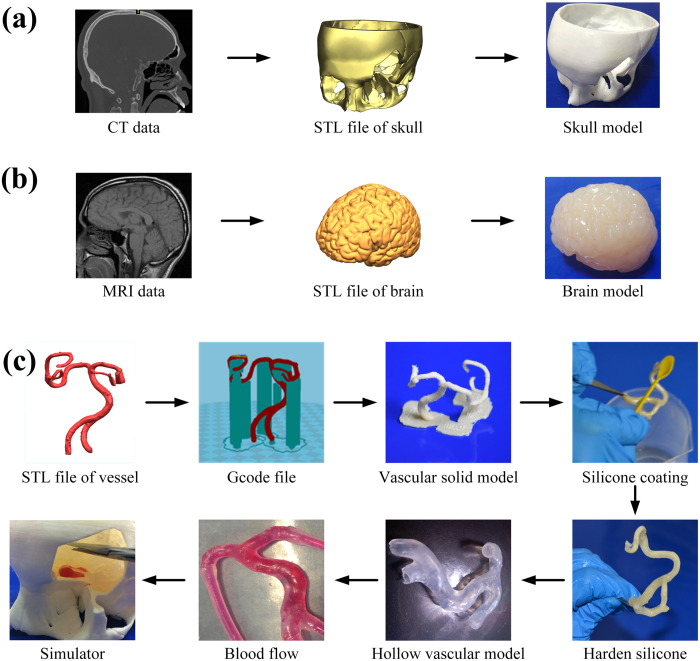
Fabrication process of a cerebral aneurysm simulator. (**a**) Fabricating a skull model by 3D printing; (**b**) Fabricating a brain model by 3D printing and silicone casting techniques; (**c**) Fabricating a vascular model with blister-like dilation bulges by 3D printing and coating techniques.

As shown in Fig. 1a, the skull model was directly 3D printed according to STL file which was converted from CT dataset. And as shown in Fig. 1b, the brain model was fabricated using a desktop 3D printer combined with silicone casting technology, in which a brain mold cavity was first 3D printed according to MRI dataset and then casted with silicone.

Further, as shown in Fig. 1c, the vascular model was fabricated using a desktop 3D printer combined with silicone coating technology, in which a vascular solid model was first 3D printed according to CT dataset and then coated with silicone. In this process, we painted low-hardness silicone on the specified surface of the vascular solid model and high-hardness silicone on the others in order to get a blister-like dilation bulge on the vessel. The vascular model was immersed in water after the silicone coating of the vascular model became completely hardened. The PVA model and xylitol coating could melt into water. A hollow elastic vascular model with a softer spot was obtained after washing the remaining PVA. Finally, an integrated cerebral aneurysm simulator was got by assembling the above fabricated skull model, brain model and vascular model.

### Fabrication of cerebral aneurysm with sequential blood flow

**Figure 2:**
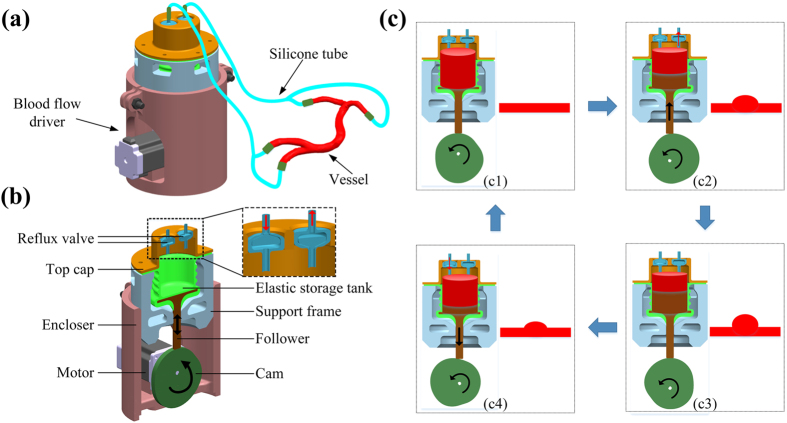
Project of the blood flow driver. (**a**) The concept map of the blood flow driver; (**b**) The physical cutaway view of the blood flow driver; (**c**) The principle and detail working process of the blood flow driver.

In order to simulate blood flow under pulsation pressure and offer motivation to form blister-like dilation bulges on the vascular model, a simple blood flow driver was fabricated. The concept map of the blood flow driver is shown in Fig. 2a,b. The blood flow driver was filled with fluid. As cam rotating, the follower is exactly driven to produce desired motion. We change rotate speed of a motor by adjusting resistor value, which imitate heart rate. Initial pressure of blood flow can be controlled by adjusting the relative position of the support frame and encloser which are fabricated by 3D printing. The regularity recreates the clinical blood flow and pulsation pressure which result in pressure waveforms in the vascular model of the simulator similar to the human.

The detail working process of the blood flow driver is shown in Fig. 2c. The cam mechanism driven by motor makes the volume of the elastic storage tank change regularly. The maximal cam lift *h* maximum is 12 mm. The quantitative information for the blood flow is shown in Table 1. The sequential blood flow then passes through the hollow elastic vascular model with regularity. The whole working process of the sequential blood flow driver can be divided into four steps. First of all, the follower locates in the cam angle for inner dwell and the lift *h* is zero. As the elastic storage tank has reached saturation, no fluid flows out or flows into the reflux valves. There is no blister-like dilation in vascular model. In the second step, as cam rotating, the follower is exactly driven to the cam angle for rise and the lift *h* is on the increase. The fluid that flows out from one of the two reflux valves with the volume of the elastic storage tank is on the decrease. The blister-like dilation in vascular model appears and starts to get bigger with cam rotating. In the third step, the follower is exactly driven to the cam angle for outer dwell and the lift *h* reaches its maximum. No fluid flow out or flow into the reflux valves. The volume of the elastic storage tank reaches its minimum, but the volume of the blister-like dilation in vascular model reaches its maximum. In the last step, the follower is exactly driven to the cam angle for return and the lift *h* is on the decrease. The fluid that flows into one of the two reflux valves with the volume of the elastic storage tank is on the increase. The blister-like dilation in vascular model starts to get smaller with cam rotating. After finishing the last step, the follower will be driven to the first step and repeat the designed cycle.

### Fabrication of cerebral aneurysm with dynamic bulging process

**Figure 3:**
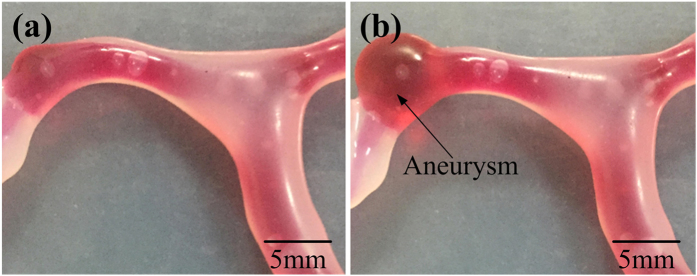
Comparison diagram of the vascular model. (**a**) No blister-like dilation bulges in vascular model with the lift of the follower *h* is zero; (**b**)Vasculature bulges a blister-like dilation similar to aneurysm.

The 3D vascular solid model is fabricated by 3D printer. This is based on magnetic resonance images and computed tomography. The dicom files are successfully converted into STL files using mimics. The vascular solid model is created by painting low-hardness silicone which can make a weak spot on some small surface and high-hardness silicone on others. At last, the vascular model was immersed in water and we create a hollow, elastic vascular model. We used a variable resistor switch to control the blood flow driver which is created to build blood flow and pulsation pressure of the hollow vascular model. As the follower rotates to the cam angle for inner dwell and the lift *h* is zero, there is no blister-like dilation in vascular model, which is shown in Fig. 3a. With the cam rotating, the blood flow driver puts fluid pressure on blood flow. When pulsating liquid flow into vascular model, preconcerted blister-like dilation appears in branch. As is shown in Fig. 3b, vasculature bulges a blister-like dilation similar to aneurysm which is a weak spot on a blood vessel. The pulsation pressure of blood flow controls the size of the dilation. Rotational speed of the cam controls the pulsatile frequency of the dilation. Blister-like dilations with maximum diameters ranging from 5 to 10 mm were successfully produced (see the supplementary video).

**Figure 4:**
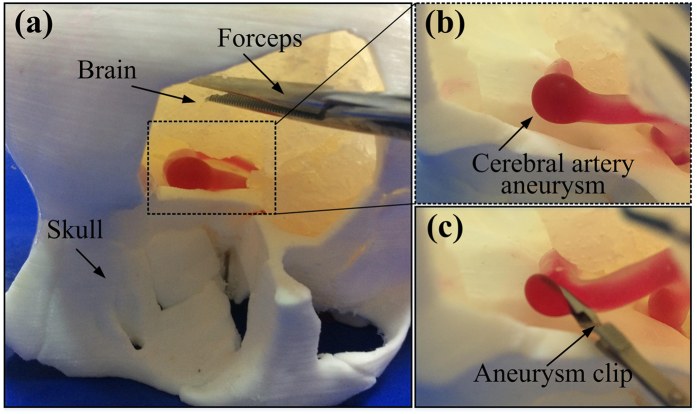
Performing aneurysm surgery training under simulated conditions. (**a**) The cerebral aneurysm simulator integration of the vascular model with a blister-like dilation bulging, a skull model and a brain model; (**b**) A magnified photograph of cerebral aneurysm in the simulator; (**c**) A magnified photograph of a resident applying an aneurysm clip.

We successfully build an anatomically personalized cerebral aneurysm simulator by the proposed method for simulating aneurysm which is shown in Fig. 4a. Brain, skull, and vascular were assembled together to locate and visualize cerebral aneurysms which is crucial to the young surgeon training. The designed simulator showed its capability of delivering the 3D morphology of an aneurysm as well as its connection with neighboring vasculature. Particularly, it is of critical importance for surgeons to precisely locate the aneurysm and thoroughly clarify its positional relationship with neighboring arteries, brain and skull. A full understanding has to be gained of the hidden structures from different viewing angles. Skull and brain offered the trainees with surgical landmarks, but as well as blocked surgeon’s field of view somehow in real operation. Clinical blood flow and pulsation pressure are recreated through silicon tubes regularity linking the blood flow driver. Within the brain and skull, the vascular model and related silicone tubes got assembled in anatomically-correct positions. The blister-like dilation bulged in vasculature is located and magnified, as demonstrated in Fig. 4b. The designed simulator (incl. skull, brain and vascular model) reproduce the field of view and the working space in aneurysm surgery.

We apply aneurysm clips to perform mock surgical interventions included in the proposed simulator and occlude the blister-like dilation similar to aneurysm included in the vascular model as shown in Fig. 4c. Regarding to anatomic awareness and improving surgical skill, the proposed simulator deserves to be an effective surgical simulator. According to the accurate anatomic scale, the proposed skull, brain, and vascular model were made. This geometric accuracy creates a nearly realistic circumstance in which the trainee can perform standard surgery training. And during a surgical intervention, the appreciation of what may be observed would be gained.

### Fabrication of vascular model with controllable properties

**Figure 5:**
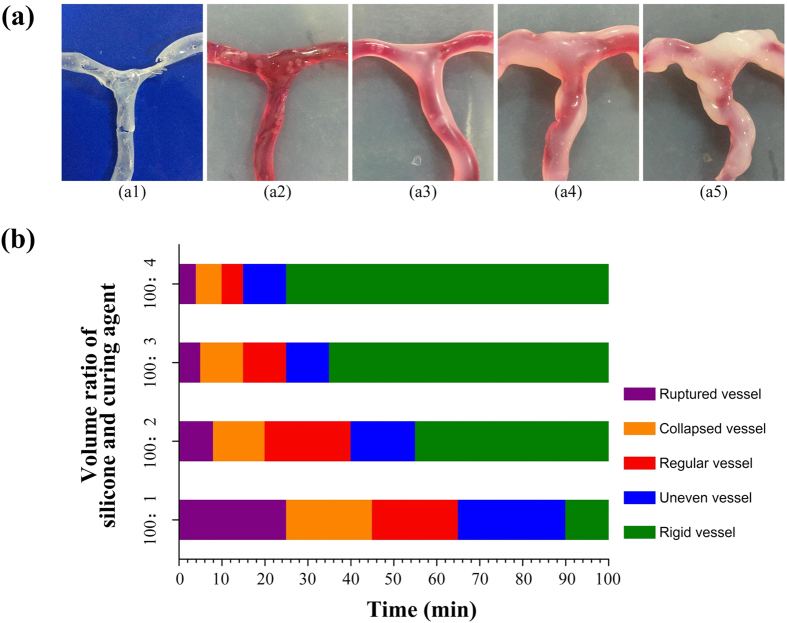
Vascular model fabrication analysis. (**a**) Five types of vascular model; (**b**) Data of the volume ratio of silicone and curing agent and time.

In the cerebral aneurysm simulator, the hollow vascular model fabrication is crucial since we simulate the formation process of cerebral aneurysm and dynamic blister-like dilation process. The shape of vascular model will affect dynamic blister-like dilation process. We paint silicone on the surface of the vascular model. So we make systematic study on both factors that will affect the shape of vascular model. The both factors are the curing time of silicone and the ratio of curing agent which is mixed with silicone. In this study, there are five types of vascular model which is shown in Fig. 5a. They are respectively ruptured vessel, collapsed vessel, regular vessel, uneven vessel and rigid vessel. For example, as shown in Fig. 5b, in this case whose volume ratio silicone and curing agent is 100:2, ruptured vessel is made below 8 minutes curing time of silicone after mixture. Collapsed vessel is made between 8–20 minutes. Regular vessel is made between 20–40 minutes. Uneven vessel is made between 40–55 minutes. Rigid vessel is made beyond 55 minutes. We can derive conclusions from quantitative data that the time interval with the volume ratio silicone and curing agent increasing in the first four types of vascular model is decreasing. But the time interval of the rigid vessel is increasing which means the curing rate of silicone is faster with the volume ratio silicone and curing agent increasing.

**Figure 6:**
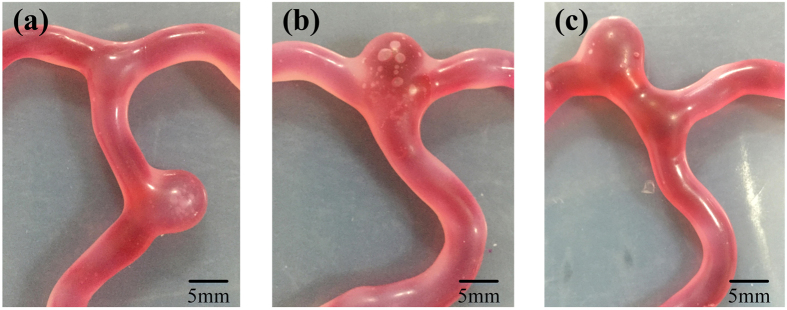
Blister-like dilations analysis. (**a**) The blister-like dilation located at the trunk of the vascular model; (**b**) The blister-like dilation located at the root of the branch; (**c**) The blister-like dilation located at the left branch.

We propose simulate dynamic blister-like dilation process and the simulator is suited to different patients. Cerebral aneurysm can attack patients in different position of the cerebral artery vessels. The three-dimensional cerebral aneurysm model that was established in different position of the same cerebral artery vessel by the proposed method is useful in assisting morphology study and guiding clinical work. The three-dimensional vascular solid model can be fabricated after removing mechanically the base and supports by using 3D printer. And we print some vascular solid models which are in the same shape. We paint the mixture low-hardness silicone evenly on the surface of the vascular model in different position. As is shown in Fig. 6, the blister-like dilations of the same anatomical vascular models but in different places are successfully created. The first blister-like dilation locates at the trunk of the vascular model. The second blister-like dilation locates at the root of the branch. And the third locates at the left branch. The simulator includes multiple blister-like dilations filled with blood flow as encountering in clinical practice. A single simulator allowing to simulate different conditions can be used in many of the aneurysms.

### Cost analysis

The major benefits of cerebral aneurysm simulator lie in the simplicity of operation and low cost for all needed instruments materials. Silicone remains as the most costly material with its softness, strength and persistent stability to be generally adopted for silicone model casting and silicone coating painting. The cost of the model and mold fabrication is very economic since they can be simply printed with a desktop 3D printer for cyclic utilization. The fabrication of another new brain model could only cost 720g silicone after having printing the brain mold and removing the base and supports. Other materials, apparatus or reagents needed for the simulator all of low cost and accessible. Table 2 has revealed that the cost could be as low as $22.88 for first-time fabrication of the entire cerebral aneurysm simulator.

A model and a casting mold could be printed in variety of materials by almost all 3D printers offered in the market, such as SLS, SLA or inkjet. Generally, SLS and SLA printers are competitive in delivering high resolution and the surface roughness of the model and casting mold fabricated by them could be managed within 10μm. However, pricing over $200,000 sometimes might sound too expensive. Inkjet 3D printers actually cost much less as they use gypsum power, which, however, requires a further processing after printing. Unlike these three, FDM remains an ideal option considering its dramatically low cost and fine surface quality produced. PLA was adopted by us to print our models and mold. Generally, this material warps consistently less than others do. Besides, it is also an economic material and contains fewer split layers if accelerating the printing. PLA is not only primarily used by us, but also widely adopted due to its flexibility in whether to use heated-build plate or not. The desktop 3D printer above applies 1.75mm diameter filament. A 1kg spool of 1.75mm PLA filament costs $11.90 and a 1kg spool of 1.75mm PVA filament costs $21.70 at the time of writing.

### Simulating clip occluding

**Figure 7:**
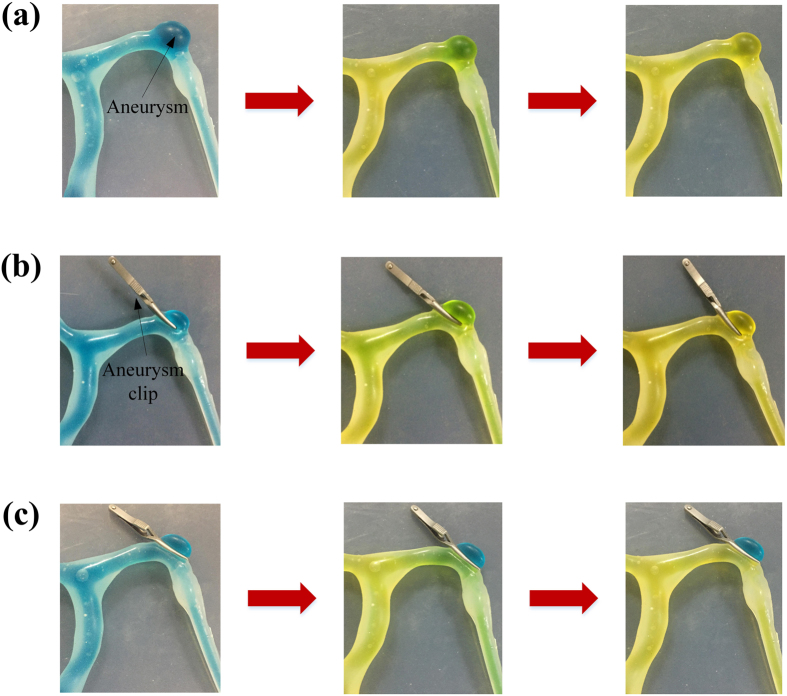
Simulating clip occluding. (**a**) The vascular model without aneurysm clips occluding the blister-like dilations; (**b**) Using aneurysm clips to occlude two thirds of the blister-like dilation, leaving a small hole; (**c**) Applying aneurysm clips to occlude the whole blister-like dilation.

We successfully created blister-like dilation with the proposed method and apply aneurysm clips to occlude the blister-like dilation, but we don’t know how evaluate the process of simulation. The quality of the proposed blister-like dilations allows a standard aneurysm clip to be realistically delivered to the aneurysm. One step of the vascular clipping is to cut off the blood flowing to the aneurysm, so it’s a common process for neurosurgeons to isolate the blood vessel feeding the aneurysm and put a small metal, clothespin-like clip on the aneurysm’s neck to halt the blood supply, with the help of microscope. So we demonstrate comparative groups to evaluate the outcome for the occluding treatment. The outcome is shown in Fig. 7. At the first, the blister-like dilation is filled with blue fluid with the blood flow driver. Then we perform three groups. At the first group we flow yellow fluid into the vascular model without aneurysm clips occluding the blister-like dilations. The fluid of the blister-like dilation’s inside changes colors from blue to green and then yellow. We apply aneurysm clips to occlude the blister-like dilation at the second group. But we deliberately use aneurysm clips to occlude two thirds of the blister-like dilation, leaving a small hole. We also can observe that the fluid of the blister-like dilation’s inside changes colors from blue to green and then yellow. The simulation of the second group shows the failing case that applied aneurysm clips to occlude the blister-like dilation. At the third group, we apply aneurysm clips to occlude the whole blister-like dilation. After finishing occluding the blister-like dilation, we start flow yellow fluid into the vascular model. The fluid of the vascular inside changes colors from blue to green and then yellow. But the fluid of the blister-like dilation’s inside don’t change the color which is blue. The simulation of the third group shows the successful case which has profound importance.

**Figure 8:**
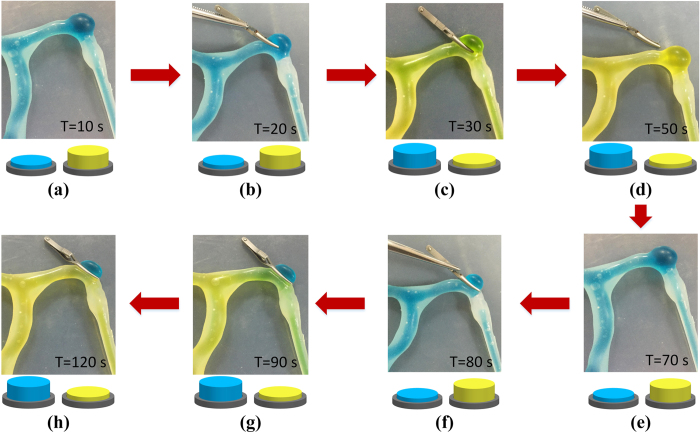
The resident apply aneurysm clip to occlude cerebral aneurysm with changing fluid marked different color by controlling on-off.

The aneurysm clips occluding the aneurysm could be simulated in three basic ways, cadaveric tissue model (animal or human) as a primordial way, along with virtual reality or computer-based systems. Now, synthetic physical models are developed as the latest approach. All of these methods have their pros and cons based on the application in the past neurosurgical simulation. To simulate the aneurysm clipping, the simulator we designed is supposed to show the greatest potential in offering training residents with an inexpensive, effective and dynamic method. For example, as is shown in Fig. 8, the simulation of the second group shows the failing case which is a resident’s training. During the simulating process, the resident can change fluid marked different color by controlling on-off. On 10 seconds, flowing yellow fluid into the vascular model and starting apply aneurysm clip to occlude the blister-like dilation under the condition. On 30 seconds, sign of the simulation shows fail by switching from blue fluid to yellow. Removing the clip from the dilation and simulating again. Switching from yellow fluid to blue on 70 seconds and applying aneurysm clip to occlude the dilation on 80 seconds. On 120 seconds, sign of the simulation shows success by switching from blue fluid to yellow, where the color of fluid flowing into the vascular model shows yellow but blue of the blister-like dilation. And the resident can simulate aneurysm clips to occlude the aneurysm repeatedly until success. In this study, the simulator was trialed by some medical students (n = 4) and neurosurgeons (n = 6) to qualitatively assess the simulating effects. Feedback results of the questionnaire from them are shown in Table 3, which revealed a general satisfaction with the simulator and its potential usefulness. The residents could develop a better understanding of the intracranial aneurysm and gain the experience in coping with vessel with blood flow possibly arising during surgeries through manipulating the elastic model. Particularly, it is believed that cognitive and psychomotor control development could be improved by the tactile response from the vasculature and brain, especially after multiple times of simulation. All participants agreed on the valuable potential of the training in a medical classroom. In such a case, such kind of simulation is bound to be increasingly popular for resident’s trainings.

## Conclusion

Our objective is to use a desktop 3D printer to build a cerebral aneurysm simulator in this study. The cerebral aneurysm simulator developed by us has facilitated the training of resident during gaining practices of performing aneurysm surgeries. Surgeons are offered with a better 3D understanding of cerebral aneurysm rather than a simple simulated 3D display. To enable the flowing within the vascular model, we’ve also developed related techniques. Not only being easy to operate and economic, the simulator fabrication method could produce a cerebral aneurysm simulator with great potential in the educational application, medical assessment and even surgical planning. The simulator allows the creation of dynamic medical scenarios with no need to worrying about the patient care. It is worth more effort in the future to improve the simulator to cover more of the cerebral vasculature and validate the overall simulator to help residents developing their skills.

## Methods

### Materials

In this study, a common fused deposition modeling (FDM) as our desktop 3D printer with a price of about $600.00 (D-Force 400, Trianglelab Co., Ltd., Jiangsu, China) was used to print all parts of the simulator. A biodegradable and bioactive thermoplastic aliphatic polyester derived from renewable resources was used for polylactic acid (PLA) filaments with a density of ρ = 1.24~1.26 g/cm^3^, a usually extruded printing temperature of 190~220 °C, a flexure strength of 50~105 MPa, a tensile strength of 30~76 MPa, a breaking elongation of 3.0~8.6% at a room temperature of 23 °C. The flow rate is 0.15~3.6 g/min (190 °C) and 0.6~7.8 g/min (210 °C). PLA is insoluble in water. It is soluble in chlorinated solvents, hot benzene, tetrahydrofuran, and dioxane. It is printed on a heated build platform at around 50 °C. A water-soluble synthetic polymer was used for polyvinyl alcohol (PVA) filaments with a density of ρ = 1.19~1.31 g/cm^3^, a usually extruded printing temperature of 190~210 °C, a flexure strength of 30~100 MPa, a tensile strength of 20~65 MPa, a breaking elongation of 11.0~17.2% at a room temperature of 23 °C. The flow rate is 0.10~2.4 g/min (190 °C) and 1.8~6.2 g/min (210 °C). It is printed on a heated build platform at around 60 °C. The average rate of PVA solubility in 65~75 °C with no source of agitation was 0.002 g/min. PLA with a 1.75 ± 0.05 mm filament diameter and ± 0.07 mm filament roundness (HORI Co., LTD., Beijing, China) and PVA with a 1.75 ± 0.05 mm filament diameter and ± 0. 07 mm filament roundness (HORI Co., LTD., Beijing, China) were used as the printing materials. Skull model and brain mold were printed with the PLA filaments. The brain mold was casted with silicone (Zhuhai COCA New Materials Co., LTD, Guangdong, China) which are mixed part A and part B in a 100:2 (volume: volume) ratio. The density of silicone is 1.12 g/cm^3^. The viscosity of silicone is 4000–5000 cs. Reducing hardness and strength by adding silicone oil into mixed silicone. The low hardness of silicone model ranges from 1 to 2 A° and the high hardness ranges from 4 to 6 A°. Vascular solid model was printed with the PVA filaments. Mixture silicone was painted evenly on the surface of the solid vascular model to fabricate vascular model. In this simulator, a blood flow driver was used for building blood flow and pulsation pressure. Some parts of the driver were printed by using PLA. The driver has a motor as its driving force. Reflux valves (Guangzhou tetrafluorohydrazine products Co., LTD, Guangdong, China) and silicone tubes (Kamoer Fluid Technology Co., LTD., Shanghai, China) were units of the driver. Red, blue and yellow dye solutions were used for the flow fluid.

### Fabricating cerebral aneurysm simulator

A patient’s head computed tomography (CT) dataset was used to construct the corresponding skull model. First, clinical neuroimaging Digital Imaging and Communications in Medicine (DICOM) images from the radiology department was obtained. Once acquired, the datasets were imported into mimics (Materialise, Leuven, Belgium), a medical reconstruction software suite. Image intensity was used to separate bone from non-bone and exported the 3D surface as an STL file (Stereo Lithography; surface data as an aggregation of fine triangular meshes). Subsequently, the STL file was converted into G code file by a slicing software tool, which can be read by a desktop 3D printer. After generating G code, it was printed.

The corresponding brain model was created using a patient’s brain magnetic resonance imaging (MRI) dataset. Clinical neuroimaging Digital Imaging and Communications in Medicine (DICOM) images from the radiology department was obtained. Then, the dataset was imported into Mimics and segmented. The brain surface model into an STL file was converted. The resulting standard 3D file format was then imported into Magics (Materialise, Leuven, Belgium), an engineering software suite. We partitioned the brain surface and created STL files of the left and right cerebral hemispheres. Subsequently, the STL files were converted into G code files for printing. After printing, commercially available molding silicone part A and part B were mixed in a 100:2(volume: volume) ratio and was poured into the brain silicone molds. The silicone was colored skin color. We extracted the brain from the mold after the silicone became completely hardened (6 A°). Then the brain was extracted from the mold after the silicone became completely hardened about 6 hours at room temperature.

The vascular computational model was fabricated using patient’s angiography CT datasets. We import computed tomography angiography datasets into Mimics and then convert results into an STL file. The vascular meshes were imported into Magics, in which we can synthesize vascular meshes into a single computational model. It was feasible for vascular branches as small as 1 mm in diameter by 3D printing. The solid, sacrificial vascular tree was 3D printed with PVA filaments.

Spicules of the vascular solid model were removed with a knife. The model is based on computed tomography and magnetic resonance images. The comparison between vascular computational model and the printing model characteristics that is shown in Table 4 was to assess the geometric accuracy of the model and the potential use for simulations. Vascular computational models with diameters ranging from 3 to 6 mm were successfully printed. Melting xylitol was smeared on the vascular solid model. It can improve the smoothness of the inner wall of the vascular model and the model’s soluble rate. We use commercially available molding silicone which is colored translucence. The silicone part A and part B were mixed in a 100:2 (volume: volume) ratio. The mixture silicone were painted evenly (0.6~0.8 mm thick) on the surface of the PVA model. Since we simulate the formation process of cerebral aneurysm and dynamic bulging process, hardness of the used silicone is different. We paint low-hardness silicone (1~2 A°) which can make a weak spot on some small surface of the vascular model and paint high-hardness (4~6 A°) silicone on the others. After about 5~6 hours at room temperature, the silicone coating of the model became completely hardened. Then the model and xylitol coating was immersed in warm water that can accelerate melting rate in 6~10 hours. At last we create a hollow, elastic vascular model.

When generating g code, basic parameters to determine how will print the model, such as layer height or print speed were set. The basic parameters of skull model, brain mold are shown in the following Table 5, and the basic parameters of vascular solid model are shown in the following Table 6. A typical FDM 3D printer, was used to print skull model, brain mold and vascular solid model in two kinds of composite materials. This 3D printer is an industrial grade configuration measuring 560 mm × 650 mm × 1300 mm and weighing 35 kg. The maximum size of fabrication is 280 mm × 280 mm × 400 mm that meets the requirement of the model’s maximum dimensions of 180 mm × 200 mm × 240 mm. It prints solid models by injecting PLA/PVA melted at 200 °C from a nozzle to repeatedly draw 0.2 mm thick pattern layers on a heated platform. The models are printed together with automatically printed base and supports.

Following finishing printing and cleaning base and supports, the model’s maximum dimensions in three defined axes were measured by using caliper to compare the print models size with the patient’s original imaging. As is shown in Table 7, the size of the final model is given along three axes: anterior to posterior (A-P), right-left (R-L), and top-bottom (T-B). Silicone curing process is actually the cross-linking density increasing process. The cross-linking density can be external shown the silicone hardness. So we measure the hardness of silicone to study cross-linking degree. The mechanical and physical properties of vascular silicone model are shown in Table 8.

### Fabricating blood flow driver

The blood flow driver is divided into two parts: execution module and control module. The execution module consists of three main parts, including cam mechanism, an elastic storage tank and two reflux valves. The cam and its follower are fabricated by 3D printing. The cam is designed according to the heart beat curve. In each kind of situation, when cam rotation, with cam contact another components named from follower, also about carries on high and low movement. The elastic container whose mold is fabricated by 3D printing is casted by silicone. And the elastic storage tank whose volume can be varied imitates left atrium and ventricle. One of the reflux valves which are attached in top cap only can inflow and the other only can outflow. These imitate mechanical mitral and aortic valves. As for the control module, we used a variable resistor switch to control a motor, which was used to drive cam mechanism. For these 3D printed parts, like a cam, a support frame and an encloser, we designed the 3D model of these parts by a typical 3D modeling software like UG NX. The 3D model was saved as a STL file and was imported into printing software, Cure, in which the G code was generated. Then we use fused deposition modeling (FDM) as our desktop 3D printer following the G code to print these parts, with the use of PLA. The blood flow driver and the hollow elastic vascular model are connected through silicone tubes.

## Additional Information

**How to cite this article:** Liu, Y. *et al*. Fabrication of cerebral aneurysm simulator with a desktop 3D printer. *Sci. Rep.* **7**, 44301; doi: 10.1038/srep44301 (2017).

**Publisher's note:** Springer Nature remains neutral with regard to jurisdictional claims in published maps and institutional affiliations.

## Supplementary Material

Supplementary Video

Supplementary Information

## Figures and Tables

**Table 1:** Quantitative information for the blood flow.

	Heart Rate	Stroke Volum (ml)	Pressure (mmHg)	Reynolds number Re	Tube diameter (mm)
Value	75	20.0	0-110.0	665.7	4.8

**Table 2:** The fabrication cost of cerebral Aneurysm Simulator for first timers.

Item	Amount	Cost
Silicone	785.70 g	¥110.00
PLA	385.50 g	¥19.30
PVA	5.20 g	¥0.92
Xylitol	1.00 g	¥0.12
Motor	1.00	¥20.00
Electric Charge	≈0.10 kW·h	¥0.05
Silicone Tube	30.00 cm	¥0.15
Reflux Valve	2.00	¥2.40
Dye Solutions	0.10 ml	¥0.01
Total		¥152.95/$22.88

**Table 3:** Feedback results.

	Satisfaction (%)
Size of the Simulator	100%
Haptic Anatomy of the Simulator	80%
Visual Appearance of the Simulator	90%
Teaching	100%
Learning	100%
Surgical Training	90%
Improving Understand the Structure of the Aneurysm’s relationship to the Parent Artery	90%
Would You Use the Simulator?	100%

**Table 4:** Vascular computational model and the printing model characteristics.

	Computational Model Volume (mm^3^)	Printing Model Volume (mm^3^)	Index of Similarity (%)
Model 1	1616.9	1529.6	94.6%
Model 2	1085.5	1034.5	95.3%
Model 3	1052.2	998.6	94.9%
Model 4	896.0	868.3	96.9%

**Table 5:** Basic parameters of skull model and brain mold.

Parameters	Value
Nozzle Size (mm)	0.4
Layer Height (mm)	0.2
Shell Thickness (mm)	0.8
Bottom/Top Thickness (mm)	0.2
Skull Model Fill Density (%)	70
Brain Mold Fill Density (%)	50
Print Speed (mm/s)	60
Printing Temperature (°C)	200
Bed Temperature (°C)	50
Diameter (mm)	1.75
Flow (%)	100

**Table 6:** Basic parameters of vascular solid model.

Parameters	Value
Nozzle Size (mm)	0.4
Layer Height (mm)	0.2
Shell Thickness (mm)	0.8
Bottom/Top Thickness (mm)	0.2
Fill Density (%)	100
Print Speed (mm/s)	45
Printing Temperature (°C)	200
Bed Temperature (°C)	60
Diameter (mm)	1.75
Flow (%)	100

**Table 7:** Measurements of the final model.

	A-P (mm)	R-L (mm)	T-B (mm)
Skull			
CT 1	179.4	151.2	183.5
Model 1	179.7	151.6	183.9
CT 2	194.1	144.0	180.8
Model 1	194.5	144.4	181.3
Brain			
MRI 1	147.8	128.4	113.6
Model 2	148.1	128.0	114.1
MRI 1	172.1	129.8	115.2
Model 1	172.5	130.6	114.6

**Table 8:** Mechanical and physical properties of silicone model.

Volume ratio of silicone and curing agent	100:1	100:2	100:3	100:4
Percentage Elongation (%)	490	470	460	470
Percentage Shrinkage (%)	1.1	1.6	2.3	2.6
Tear Strength (kN/m)	7.0	7.1	7.2	7.1
Tensile Strength (MPa)	2.0	2.1	2.2	2.1
Hardness (A°)	4.5	5.0	5.5	5.0
Heat Resistance (°C )	200–300	200–300	200–300	200–300
