# Supplementary material for: Fabrication of cerebral aneurysm simulator with a desktop 3D printer
Source: Sci Rep. 2017 May 17;7:44301. doi: 10.1038/srep44301 (PMC5434791; doi:10.1038/srep44301)
Supplement: Supplementary Information [file srep44301-s2.pdf]

Supplementary Information

Fabrication of cerebral aneurysm simulator with a desktop 3D printer

Yu Liu[1,2,‡], Qing Gao[1,2,‡], Song Du[3], ZiChen Chen[1], JianZhong Fu[1,2], Bing Chen[4*],

ZhenJie Liu[4], Yong He[1,2*]

(1. State Key Laboratory of Fluid Power and Mechatronic Systems, School of Mechanical Engineering, Zhejiang University, Hangzhou 310027, China

2. Key Laboratory of 3D Printing Process and Equipment of Zhejiang Province, School of Mechanical Engineering, Zhejiang University, Hangzhou 310027, China

‡These two authors contributed equally to this work.

3. Department of Neurosurgery, Second Affiliated Hospital, School of Medicine, Zhejiang University, Hangzhou 310009, China.

4. Department of Vascular Surgery, Second Affiliated Hospital, School of Medicine, Zhejiang University, Hangzhou, 310009, China

**Correspondence to: Bing Chen, e-mail: cbobc@163.com or Yong He, e-mail: yongqin@zju.edu.cn)*

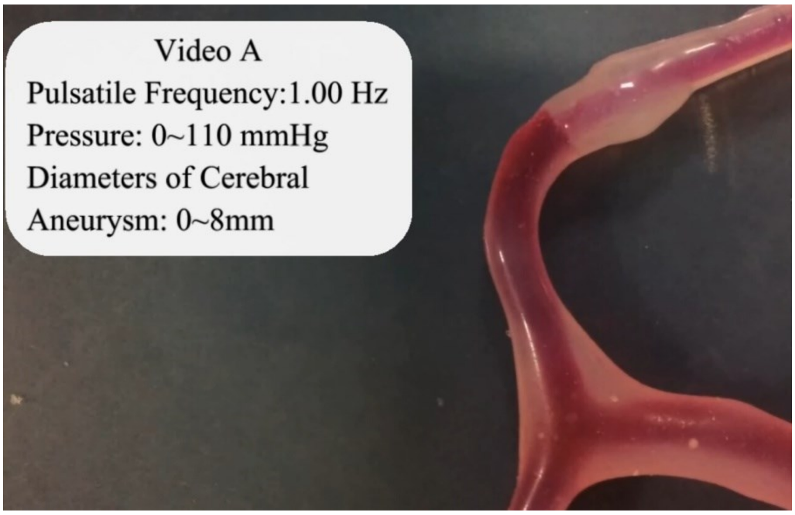

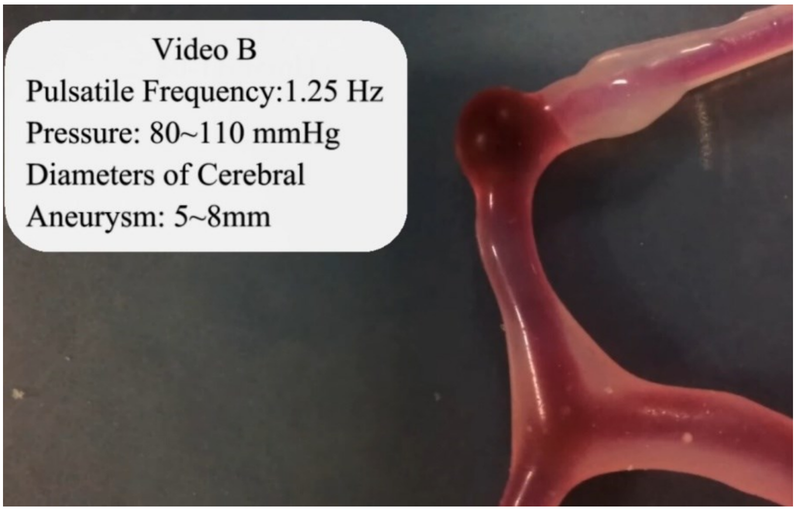

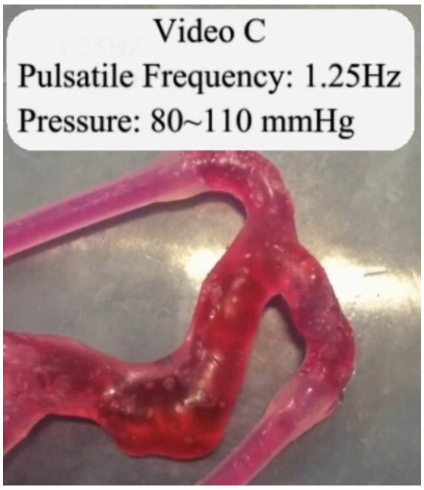

Supplementary Video. (see **Supplementary_Video.avi** for the entire video)
The pulsatile frequency and the maximum diameters of cerebral aneurysm: In the video A, the diameters of cerebral aneurysm range from 0~8 mm with the conditions that the pulsatile frequency of 1Hz and pressure of 0~110 mmHg. In the video B, the diameters of cerebral aneurysm range from 5~8 mm with the conditions that the pulsatile frequency of 1.25Hz and pressure of 80~110 mmHg. The blister-like dilation in vascular model of the video C is irregular with the conditions that the pulsatile frequency of 1.25Hz and pressure of 80~110 mmHg.
